# CAFR-Net: A transformer-contrastive framework for robust spinal MRI segmentation via global-local synergy

**DOI:** 10.1371/journal.pone.0327642

**Published:** 2025-07-17

**Authors:** Rui Ma, Xuegang Dai, Zuochao Yang, Zhixiong Wei, Bin Zhang

**Affiliations:** Information Center, Gansu Provincial Maternity and Child care Hospital (GansuProvincial Central Hospital), Gansu, China; Shijiazhuang Tiedao University, CHINA

## Abstract

Automated spinal structure segmentation in sagittal MRI remains a non-trivial task due to high inter-patient variability and ambiguous anatomical boundaries. We propose CAFR-Net, a Transformer-contrastive hybrid framework that jointly models global semantic relations and local anatomical priors to enable precise multi-class segmentation. The architecture integrates (1) a multi-scale Transformer encoder for long-range dependency modeling, (2) a Locally Adaptive Feature Recalibration (LAFR) module that reweights feature responses across spatial-channel dimensions, and (3) a Contrastive Learning-based Regularization (CLR) scheme enforcing pixel-level semantic alignment. Evaluated on the SpineMRI dataset, CAFR-Net achieves state-of-the-art performance, surpassing prior methods by a significant margin in Dice (92.04%), HD (3.52 mm), and mIoU (89.31%). These results underscore the framework’s potential as a generalizable and reproducible solution for clinical-grade spinal image analysis.

## 1 Introduction

Spinal magnetic resonance imaging (MRI) plays a pivotal role in the diagnosis and treatment planning of spinal disorders, including degenerative diseases, trauma-induced lesions, and neoplasms. Its superior soft-tissue contrast and non-invasive nature make it the modality of choice for visualizing the complex anatomy of the spine. Accurate segmentation of spinal structures—particularly vertebral bodies, intervertebral discs, and the spinal cord—is essential for quantitative analysis, surgical planning, and longitudinal disease monitoring. However, achieving precise and robust segmentation in clinical settings remains highly challenging due to multiple intrinsic and extrinsic factors [1, 2].

First, the inherent low contrast between adjacent anatomical tissues in spinal MRI leads to weak and ambiguous boundaries, particularly between intervertebral discs and vertebrae. Second, pathological conditions such as disc herniation, spinal canal stenosis, and vertebral degeneration introduce substantial morphological variability and localized structural deformations. Third, inter-subject differences in spinal curvature, vertebral shape, and disc spacing add further complexity, making the segmentation task highly non-trivial and data-sensitive [3, 4].

Convolutional neural networks (CNNs) have shown significant success in medical image segmentation owing to their hierarchical feature representation and local receptive field mechanisms [5]. Nevertheless, their limited ability to capture long-range dependencies hampers the modeling of coherent anatomical structures across extended spatial extents, which is crucial for maintaining topological consistency in spinal segmentation [5]. Recently, Transformer-based architectures have emerged as powerful alternatives by leveraging self-attention mechanisms to explicitly model global semantic interactions [6, 7]. Despite their strength in capturing high-level representations, Transformers alone often fail to preserve spatially fine-grained details—particularly at low-contrast or ambiguous boundaries—leading to imprecise delineation of anatomical regions [8]. Moreover, these architectures typically require extensive annotated data to achieve competitive performance, posing practical challenges in medical imaging domains where high-quality labels are scarce [9].

To address the aforementioned limitations, we propose CAFR-Net, a novel Transformer-based segmentation framework specifically designed for spinal MRI. CAFR-Net integrates three complementary components to achieve both comprehensive context modeling and local structural refinement. The Transformer encoder encodes long-range semantic dependencies, forming a robust global representation. A LAFR module enhances local discriminative capability by learning spatially adaptive attention maps that selectively emphasize boundary-relevant regions. Additionally, a CLR module is introduced to enforce semantic consistency across spatially disjoint but anatomically similar regions, improving inter-class separability and intra-class compactness at the feature level.

The primary contributions of this work are as follows:

(1) We propose CAFR-Net, a unified segmentation architecture that effectively combines global semantic encoding and local feature enhancement tailored for spinal MRI.
(2) We develop a Locally Adaptive Feature Recalibration (LAFR) module that dynamically modulates spatial features to improve boundary localization in low-contrast regions.
(3) We design a Contrastive Learning-based Regularization (CLR) module that introduces feature-level semantic constraints, enhancing robustness and consistency in feature representations.
(4) We conduct comprehensive experiments on the SpineMRI dataset [24], demonstrating that CAFR-Net achieves superior segmentation accuracy over existing CNN- and Transformer-based baselines. Detailed ablation studies further validate the individual contributions of LAFR and CLR modules.

## 2 Related work

### 2.1 CNN-based segmentation

Deep learning-based approaches, particularly convolutional neural networks (CNNs), have become the dominant architecture for medical image segmentation [3]. U-Net [1] serves as a foundational model, with extensions such as UNet++ [10] introducing nested dense skip pathways, and Attention U-Net [11] enhancing spatial focus via attention gates. Despite their local modeling strength, CNNs suffer from limited receptive fields, which constrain their ability to capture global dependencies—especially critical in the analysis of spinal anatomy. Multi-scale networks such as ${\text{M}}^{2}$SNet [27] refine both detail and structure-level representations. Inf-Net [34] enhances segmentation in challenging scenarios with ambiguous boundaries by combining edge attention and implicit boundary supervision.

### 2.2 Transformer-based segmentation

Transformer architectures have emerged as powerful tools in vision tasks by modeling long-range dependencies through self-attention [12]. Building upon this foundation, UNETR++ [31] introduces cross-resolution attention and residual refinement modules to enhance hierarchical feature modeling for medical image segmentation. While vanilla Vision Transformers (ViTs) require large datasets and lack localization capability, hybrid models such as Swin-Unet [7] and TransUNet [6] alleviate this by combining CNN backbones with Transformer encoders. Multi-scale Transformer variants such as HiFormer [26] further enhance hierarchical context modeling across resolutions. Uformer [28] further reduces computational cost via a Unet-like structure with Transformer blocks. UNetFormer [2] follows a similar design, incorporating Transformer blocks into a UNet-like encoder-decoder architecture for efficient medical image segmentation.

Recent variants including TransFuse [16] and MCTrans [20] incorporate multi-branch or multi-scale designs to better balance spatial precision and contextual reasoning. However, these models often still struggle with fine boundary delineation and robustness under limited supervision [32]. Transformer-based segmentation has also been extended to brain tumor segmentation tasks, such as TransBTS [30], highlighting its adaptability across modalities.

### 2.3 Self-supervised representation learning

Self-supervised learning (SSL) methods aim to reduce reliance on manual annotations by leveraging pretext tasks. MAE [6] and SimCLR [22] represent two dominant paradigms: reconstruction-based and contrastive-based pretraining. DINO and BYOL [13, 19] further remove negative pairs, improving stability and scalability. In medical imaging, the recent survey by Tajbakhsh *et al*. [21] highlights both the promise and challenges of applying SSL to clinical tasks, particularly in segmentation contexts.

### 2.4 Contrastive learning for dense prediction

Conventional contrastive learning often operates at the global or patch level, insufficient for dense prediction tasks such as segmentation. SimCLR [35] introduced a simple yet powerful contrastive framework based on instance discrimination and InfoNCE loss, which laid the foundation for subsequent advances in dense prediction tasks. Chaitanya *et al*. [29] first explored the application of contrastive learning for medical image segmentation, proposing a framework to align global and local features; however, their design lacks explicit mechanisms for handling intra-organ boundary ambiguity. SegCLR [14] addresses this by introducing structure-aware contrastive objectives at the pixel level. PixContrast [17] further strengthens this direction by sampling hard positive pairs along anatomical boundaries, enhancing local discriminability. Nevertheless, most of these methods rely on spatial proximity assumptions and lack explicit modeling of semantic similarity across disjoint regions.

### 2.5 Spinal MRI segmentation

Spinal MRI segmentation presents unique challenges, including vertebral shape variation, intersubject curvature, and disease-induced structural deformations [33]. DeepSpine [4] provides a multi-task approach integrating vertebrae and disc analysis. Spine-GNN [18] leverages graph structures to encode anatomical relationships among vertebrae. Azad *et al*. [15] review the difficulties in modality-missing spine segmentation, reinforcing the need for robust, context-aware frameworks.

## 3 Method

**Fig 1:**
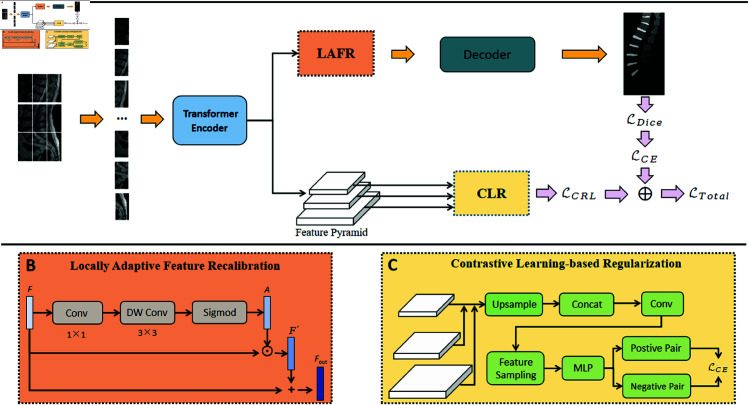
(a) Overview of CAFR-Net architecture, integrating a Transformer encoder, the LAFR module, and the CLR module. (b) LAFR module workflow: encoder features are recalibrated via 1×1 and depthwise convolutions with Sigmoid activation. (c) CLR module workflow: multi-scale features are sampled to form semantic pairs for contrastive learning via InfoNCE loss. These components collectively enhance boundary localization and semantic consistency for spinal MRI segmentation.

Our proposed CAFR-Net consists of a transformer-based encoder, a Locally Adaptive Feature Recalibration (LAFR) module, a Contrastive Learning-based Regularization (CLR) module, and a decoder for segmentation prediction. The overall framework is illustrated in Fig 1.

### 3.1 Global feature encoding via transformer

To capture long-range dependencies and global anatomical context in spinal MRI, we adopt a Transformer-based encoder as the backbone feature extractor [6, 12, 20]. Compared to conventional CNNs that are limited in modeling non-local interactions, the Transformer encoder offers stronger global context modeling while maintaining spatial granularity.

Given an input image $I\in {\mathbb{R}}^{H\times W\times C}$, we partition it into non-overlapping patches of size p×p (with *p* = 16). Each patch is flattened and linearly projected into a *D*-dimensional token:

$${𝐳}_{i}={𝐖}_{e}\mathrm{vec}({𝐏}_{i})+{𝐛}_{e},$$ (1)

where ${𝐖}_{e}\in {\mathbb{R}}^{D\times p^{2}C}$ and ${𝐛}_{e}\in {\mathbb{R}}^{D}$ are learnable parameters.

Let $𝐙\in {\mathbb{R}}^{N\times D}$ denote the embedded patch sequence, where $N=\frac{HW}{p^{2}}$. Self-attention is computed using Multi-Head Self-Attention (MHSA) [12]:

$$\text{Attention}(𝐐,𝐊,𝐕)=\mathrm{softmax}\left(\frac{{QK}^{T}}{\sqrt{D_{h}}}\right)𝐕,$$ (2)

with query, key, and value computed as:

$$𝐐={𝐖}_{Q}𝐙,\;𝐊={𝐖}_{K}𝐙,\;𝐕={𝐖}_{V}𝐙.$$ (3)

Each Transformer block follows the standard residual structure with layer normalization:

$${𝐙}^{\prime }=\mathrm{LN}\left(𝐙+\text{Attention}(𝐐,𝐊,𝐕)\right),\;{𝐙}^{\prime \prime }=\mathrm{LN}\left({𝐙}^{\prime }+\mathrm{FFN}({𝐙}^{\prime })\right),$$ (4)

where FFN denotes a two-layer feedforward network with ReLU activation.

After passing through *L* Transformer layers, the output ${𝐙}^{\prime \prime }\in {\mathbb{R}}^{N\times D}$ encodes a globally contextualized representation of the input image. This representation serves as the semantic foundation for downstream segmentation.

To facilitate scale-aware learning, we additionally extract hierarchical features from three intermediate Transformer layers at resolutions of $\frac{1}{4}$, $\frac{1}{8}$, and $\frac{1}{16}$. These features are upsampled to a unified 1/4 scale and fused via channel-wise concatenation and a 1×1 convolution. The fused representation is then utilized by the contrastive regularization module.

### 3.2 Local feature recalibration via LAFR module

To compensate for the spatial information degradation caused by patch-based tokenization in the Transformer encoder, we design a Locally Adaptive Feature Recalibration (LAFR) module. It aims to enhance spatial sensitivity by reweighting feature responses at each location in a residual manner.

Given an intermediate feature map $𝐅\in {\mathbb{R}}^{H\times W\times C}$, LAFR generates a spatially adaptive attention map through two convolutional layers. Specifically, a 1×1 convolution adjusts channel dependencies, and a depthwise 3×3 convolution captures local spatial context:

$$𝐌=\mathrm{DWConv}({\mathrm{Conv}}_{1\times 1}(𝐅)).$$ (5)

This depthwise operation enhances the model’s ability to extract localized spatial cues within each channel while maintaining computational efficiency.

The attention map $𝐀\in {\mathbb{R}}^{H\times W\times C}$ is obtained via a sigmoid activation:

𝐀=σ(𝐌). (6)

We apply element-wise multiplication to obtain the recalibrated feature:

$${𝐅}^{\prime }=𝐅⊙𝐀,$$ (7)

and fuse it with the original feature using residual addition:

$${𝐅}_{\text{out}}=𝐅+{𝐅}^{\prime }.$$ (8)

LAFR enables the network to highlight salient anatomical structures, such as vertebral boundaries and intervertebral discs, without introducing significant computational overhead.

### 3.3 Semantic consistency via contrastive learning regularization

To encourage semantic coherence across spatially disjoint but anatomically similar regions, we propose a CLR module. CLR guides the model to pull together feature embeddings from the same anatomical class while pushing apart those from different classes.

Let ${𝐟}_{i}\in {\mathbb{R}}^{d}$ denote a sampled feature from the fused multi-scale representation. Each feature is projected by an MLP and normalized to unit length. Positive and negative pairs are constructed based on ground-truth labels in the downsampled annotation map.

All contrastive pairs are constructed within each mini-batch (i.e., the current training batch). Positive pairs consist of features from the same anatomical class under different augmentations, while negative pairs are sampled from other classes at a 1:5 ratio relative to positives. To address class imbalance, we apply class-aware sampling during batch construction, ensuring adequate representation of minority classes.

For a given anchor ${𝐟}_{i}$, we define a positive sample ${𝐟}_{j}^{+}$ such that $y_{i}=y_{j}$, and negatives ${𝐟}_{k}^{-}$ where $y_{i}\neq y_{k}$.

CLR optimizes an InfoNCE-style contrastive loss:

$${ℒ}_{\text{CLR}}=-\log\frac{\exp({𝐟}_{i}\cdot {𝐟}_{j}^{+}/\tau )}{\sum_{j}\exp({𝐟}_{i}\cdot {𝐟}_{j}/\tau )},$$ (9)

where τ is the temperature hyperparameter. The dot product measures feature similarity in the embedding space.

To improve robustness, CLR operates on features from multiple spatial resolutions, ensuring semantic alignment across different receptive fields.

By enforcing contrastive consistency during training, CLR enhances intra-class compactness and inter-class separation, effectively reducing segmentation ambiguity in regions with weak boundaries or low intensity contrast.

### 3.4 Loss function and training configuration

**Loss formulation.** The overall training objective integrates region-level, pixel-level, and representation-level constraints. The total loss is defined as:

$$ℒ=\lambda_{1}{ℒ}_{\text{Dice}}+\lambda_{2}{ℒ}_{\text{CE}}+\lambda_{3}{ℒ}_{\text{CLR}},$$ (10)

where $\lambda_{1},\lambda_{2},\lambda_{3}$ are weighting coefficients.

To address class imbalance and emphasize small structures, Dice loss is employed [23]:

$${ℒ}_{\text{Dice}}=1-\frac{2\sum_{i}p_{i}g_{i}}{\sum_{i}p_{i}^{2}+\sum_{i}g_{i}^{2}}.$$ (11)

To optimize pixel-wise classification, the cross-entropy loss is applied:

$${ℒ}_{\text{CE}}=-\sum_{i}\left[g_{i}\log p_{i}+(1-g_{i})\log(1-p_{i})\right].$$ (12)

To promote feature-level semantic consistency, we incorporate the contrastive regularization loss (CLR), formulated as:

$${ℒ}_{\text{CLR}}=-\log\frac{\exp({𝐟}_{i}\cdot {𝐟}_{j}^{+}/\tau )}{\sum_{j}\exp({𝐟}_{i}\cdot {𝐟}_{j}/\tau )},$$ (13)

where τ denotes the temperature parameter and $\left({𝐟}_{i},{𝐟}_{j}^{+}\right)$ is a positive pair belonging to the same anatomical category.

To assess the robustness of the selected weighting coefficients, we conducted a sensitivity analysis by perturbing each loss weight individually while keeping the others fixed. This analysis confirmed that the chosen weights yield consistently stable segmentation performance. For detailed configurations and results, please refer to S1 Appendix.

**Training configuration.** We optimize the network using the Adam optimizer with an initial learning rate of 10^−4^, which decays to 10^−6^ following a cosine annealing schedule. Training is performed over 500 epochs with a batch size of 16. Batch normalization is applied after each convolutional block to facilitate optimization stability, and a dropout rate of 0.3 is introduced to regularize the network. A weight decay of 10^−5^ is used to enhance generalization. Early stopping with a patience of 10 epochs is employed to prevent overfitting.

The learning rate at epoch *t* follows the cosine annealing strategy, as defined in Eq 14:

$$\eta_{t}=\eta_{\min}+\frac{1}{2}(\eta_{\max}-\eta_{\min})\left(1+\cos\left(\frac{t}{T}\pi \right)\right),$$ (14)

where $\eta_{t}$ denotes the learning rate at epoch *t*.

Data augmentation strategies include random flipping and rotation to improve spatial invariance, intensity normalization to reduce scanner-induced variability, and elastic deformations to simulate anatomical diversity. These augmentations enhance generalization and robustness across subjects.

The major training hyperparameters are summarized in Table 1, providing an overview of the setup. This training strategy stabilizes optimization while preserving both global context and fine structural details, leading to improved segmentation performance in challenging spinal MRI scenarios.

**Table 1:** Summary of training hyperparameters.

Hyperparameter	Setting
Batch Size	16
Optimizer	Adam
Initial Learning Rate	$1\times 10^{-4}$
Learning Rate Decay	Cosine annealing to $1\times 10^{-6}$
Batch Normalization	After each convolutional block
Dropout Rate	0.3
Number of Epochs	500
Early Stopping	10 epochs
Weight Decay	$1\times 10^{-5}$
Data Augmentation	Random flipping, rotation, intensity normalization, elastic deformations

## 4 Experiments

To evaluate the effectiveness of CAFR-Net in spinal MRI segmentation, we conduct extensive experiments on the SpineMRI dataset[24], focusing on segmentation performance in low-contrast regions, boundary delineation accuracy, and robustness to inter-patient anatomical variability. Additionally, we perform ablation studies to analyze the contributions of key components.

### 4.1 Dataset and preprocessing

**Fig 2:**
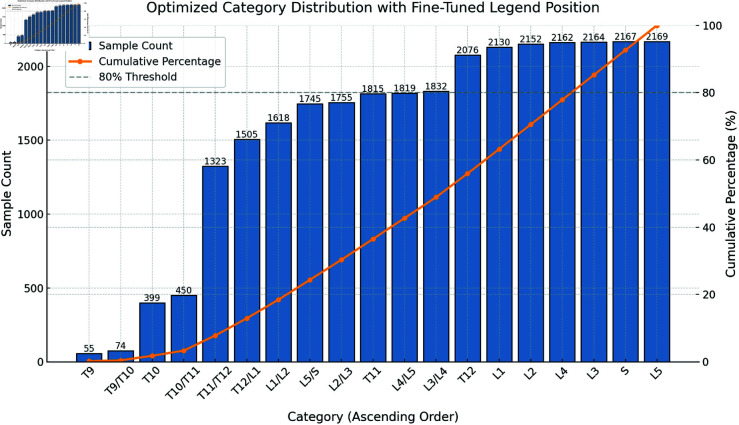
Distribution of sample counts across categories in the dataset. The bar chart represents the sample count for each category sorted in ascending order, while the orange line indicates the cumulative percentage. The 80% threshold (gray dashed line) highlights the contribution of the major categories to the overall dataset.

We evaluate CAFR-Net on the publicly available SpineMRI dataset, which comprises 172 sagittal MRI scans annotated with 19 anatomical categories, including vertebral bodies (T9–L5, S) and intervertebral discs (T9/T10–L5/S), as illustrated in Fig 2. The dataset is split into 70% for training, 15% for validation, and 15% for testing, ensuring anatomical diversity across partitions.

Spinal MRI segmentation is inherently challenging due to low inter-class contrast, irregular anatomical boundaries, and significant inter-patient variability. Moreover, class imbalance exists, as certain vertebral levels (e.g., T9–T10) are underrepresented compared to lumbar regions.

To enhance model robustness and address class imbalance, we apply a set of preprocessing and augmentation techniques. Voxel intensity normalization is performed to standardize MRI intensity values to [0,1]. To simulate inter-patient anatomical variability, we employ random rotation ($\pm 15^{\circ }$), flipping, and elastic deformations. To improve visibility in low-contrast regions, contrast-limited adaptive histogram equalization (CLAHE) and histogram equalization are applied. Additionally, a stratified patch sampling strategy ensures that underrepresented vertebral levels receive sufficient training representation. These preprocessing steps align with our model’s architectural design, ensuring consistency between training and inference conditions.

### 4.2 Experimental setup and implementation details

**Training setup and evaluation.** The proposed CAFR-Net is implemented using PyTorch and trained on an NVIDIA A100 GPU. Following the training configuration detailed in Sect 3.4, the model is optimized using the Adam optimizer with cosine annealing, a batch size of 16, and regularization techniques including dropout (rate of 0.3), weight decay ($1\;\times \;10^{-5}$), and early stopping with a patience of 10 epochs. Data augmentation strategies (including random flipping, rotation, intensity normalization, and elastic deformation) are applied as previously described.

Evaluation is performed on the SpineMRI test set using Dice coefficient (DSC), Hausdorff Distance (HD), and mean Intersection-over-Union (mIoU) as the primary metrics. A hybrid loss function combining Dice loss, cross-entropy loss, and the contrastive regularization term described in Sect 3 is employed, with loss weights set to $\lambda_{1}=0.5$, $\lambda_{2}=1.0$, and $\lambda_{3}=0.1$.

**Fig 3:**
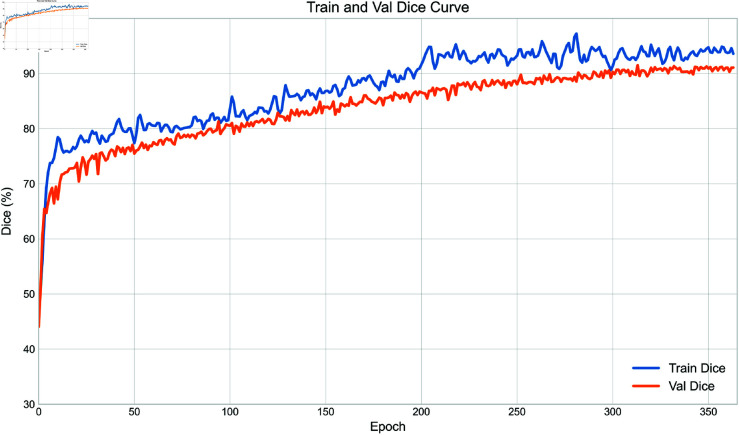
Training and validation Dice curves over 364 epochs. The consistent alignment between the two indicates stable convergence and effective overfitting control.

As shown in Fig 3, the training and validation Dice curves remain closely aligned over 364 epochs, indicating stable convergence without significant overfitting. This result supports the effectiveness of the adopted regularization strategies under limited-data conditions.

**Transformer and CLR architecture.** Each input image of size 256×256 is partitioned into 16×16 non-overlapping patches, yielding 256 tokens. Each patch is linearly embedded into a 768-dimensional token, with positional encodings added. Three shallow Transformer branches are employed to extract low-level, mid-level, and high-level features.

The CLR module adopts an InfoNCE loss with a temperature parameter of τ=0.07. Within each batch, positive pairs are formed from augmented views of the same class, while negative pairs are sampled from other classes at a 1:5 ratio. Class-aware sampling is applied to ensure equitable representation across categories. For clarity, the key hyperparameters of the Transformer encoder and CLR module are summarized in Table 2.

**Table 2:** Key architectural hyperparameters of the Transformer encoder and CLR module.

Parameter	Setting
Input Resolution	256×256
Patch Size	16×16 (256 tokens per image)
Embedding Dimension	768
Positional Encoding	Learnable
Transformer Branches	3 (low, mid, high-level)
Attention Heads per Block	6
CLR Temperature τ	0.07
Positive/Negative Ratio	1:5
Class-Aware Sampling	Applied per batch
CLR Usage Stage	Training only

### 4.3 Comparative experiments

To evaluate the effectiveness of CAFR-Net in spinal MRI segmentation, we compare it against state-of-the-art models, including U-Net [1], nnU-Net [25], Attention U-Net [11], Swin-Unet [7], and TransUNet [6]. As shown in Table 3, CAFR-Net achieves the highest DSC of 92.04%, surpassing U-Net by +6.73%, TransUNet by +3.67%, and Swin-Unet by +1.92%. Additionally, it attains the lowest HD of 3.52 mm, demonstrating superior boundary delineation, particularly in anatomically complex and low-contrast regions. The mIoU score of 89.31% further supports the model’s ability to generate high-fidelity segmentations.

**Table 3:** Performance comparison on the SpineMRI dataset [24]. DSC and mIoU measure segmentation accuracy, while HD reflects boundary precision. FLOPs are calculated with 256×256 grayscale input, batch size 1, and 20 output classes. p-values (Dice) are computed using a Wilcoxon signed-rank test comparing CAFR-Net with each baseline method.

Method	DSC (%)	HD (mm)	mIoU (%)	FLOPs	p-value (Dice)
U-Net	85.31	6.71	78.46	12.11	<0.001
nnU-Net	88.04	5.41	81.92	12.14	<0.001
Attention U-Net	87.92	5.89	81.03	12.37	<0.001
TransUNet	89.37	4.68	83.27	11.66	0.002
Swin-Unet	90.12	4.11	84.51	7.36	0.014
UNETR++	90.65	3.85	85.10	8.36	0.036
**CAFR-Net (Ours)**	**92.04**	**3.52**	**89.31**	**5.12**	–

To assess the model’s deployment feasibility in clinical settings, we further analyze its computational complexity. As shown in Table 3, CAFR-Net achieves the lowest FLOPs (5.12G) among all methods, despite integrating Transformer-based encoders and additional modules. This efficiency is primarily attributed to the use of shallow multi-scale Transformer branches, lightweight channel-wise recalibration in LAFR, and the exclusion of the CLR module during inference. These design choices allow CAFR-Net to maintain high segmentation accuracy while significantly reducing computational burden, making it suitable for time-sensitive or resource-constrained clinical scenarios. This balance of modular design and computational efficiency underscores the practicality of CAFR-Net for real-world clinical deployment.

Further analysis across different anatomical regions highlights the advantages of CAFR-Net. In low-contrast structures such as the sacrum and lower lumbar vertebrae, CAFR-Net improves Dice scores by 2.81% over U-Net and 1.57% over nnU-Net while reducing HD by 0.18 mm. These improvements can be attributed to the CLR module, which enhances feature discriminability by enforcing semantic consistency across similar anatomical regions, thereby reducing ambiguity in intensity-similar areas.

In complex boundary regions, including vertebra-disc junctions and spinal canal regions, CAFR-Net significantly improves boundary delineation, reducing HD by 0.15 mm compared to Swin-Unet and 0.12 mm compared to TransUNet. The LAFR module refines spatial feature representations, dynamically adjusting feature responses to better capture intricate boundary transitions, leading to smoother segmentations with reduced misalignment errors.

**Fig 4:**
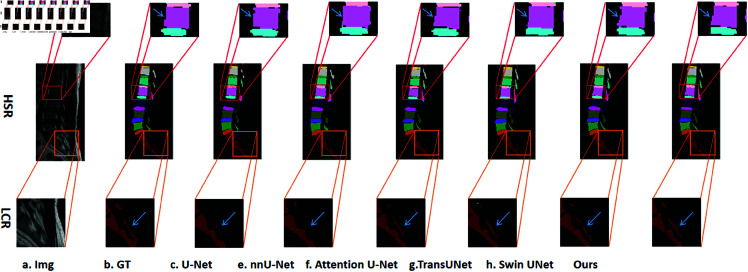
Qualitative comparison in low-contrast regions(LCR), complex boundary regions(CBR), and homogeneous signal regions(HSR). Zoomed-in boxes indicate challenging areas; blue arrows highlight key improvements made by CAFR-Net over baseline methods.

Qualitative results in Fig 4 further illustrate that CAF-RNet consistently produces more precise segmentations compared to baseline models. Unlike TransUNet and Swin-Unet, which exhibit occasional over-segmentation artifacts, CAFR-Net preserves anatomical integrity while maintaining structural consistency across samples.

**Fig 5:**
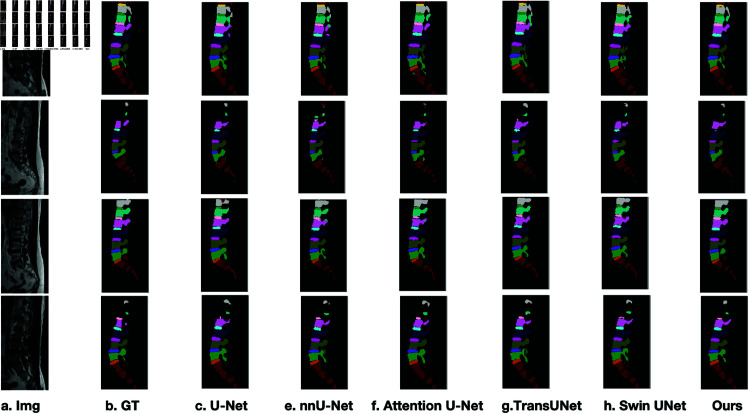
Visual comparison of segmentation results across diverse spinal MRI samples. CAFR-Net consistently achieves higher segmentation fidelity, maintaining anatomical integrity across different cases.

Beyond region-specific improvements, CAFR-Net demonstrates robust generalization across diverse spinal MRI cases. As illustrated in Fig 5, segmentation masks generated by CAFR-Net closely align with ground-truth annotations across various anatomical variations. Unlike baseline models, which often suffer from boundary fragmentation, CAFR-Net consistently preserves anatomical coherence, ensuring reliable clinical applicability.

These findings confirm that CAFR-Net effectively balances global contextual modeling with local feature recalibration, leading to state-of-the-art segmentation accuracy and superior boundary preservation. The CLR module enhances feature representation in low-contrast regions, while the LAFR module improves boundary precision, reinforcing its clinical potential in spinal MRI analysis.

### 4.4 Ablation study

To evaluate the contributions of the proposed LAFR module and CLR module, we conduct an ablation study by progressively removing these components from CAFR-Net. The results, summarized in Table 4, demonstrate the impact of each module on segmentation performance.

**Table 4:** Ablation study results on the SpineMRI dataset [24]. The full CAFR-Net model achieves the best performance across all metrics.

Model	DSC (%)	HD (mm)	mIoU (%)
Baseline	88.92	5.04	82.14
+ LAFR	90.43	4.32	84.89
+ CLR	91.12	3.98	86.01
**Full CAFR-Net**	**92.04**	**3.52**	**89.31**

The baseline model, which excludes both LAFR and CLR modules, achieves a DSC of 88.92%, indicating that while the Transformer encoder provides a strong backbone, additional refinements are necessary to enhance segmentation performance. Incorporating the LAFR module leads to a +1.51% improvement in DSC and a 0.72 mm reduction in HD, highlighting its effectiveness in refining spatial feature representations. The addition of the CLR module further enhances performance, improving DSC by +2.20% over the baseline, with an HD reduction of 1.06 mm, demonstrating its role in improving anatomical consistency and robustness to low-contrast variations.

**Fig 6:**
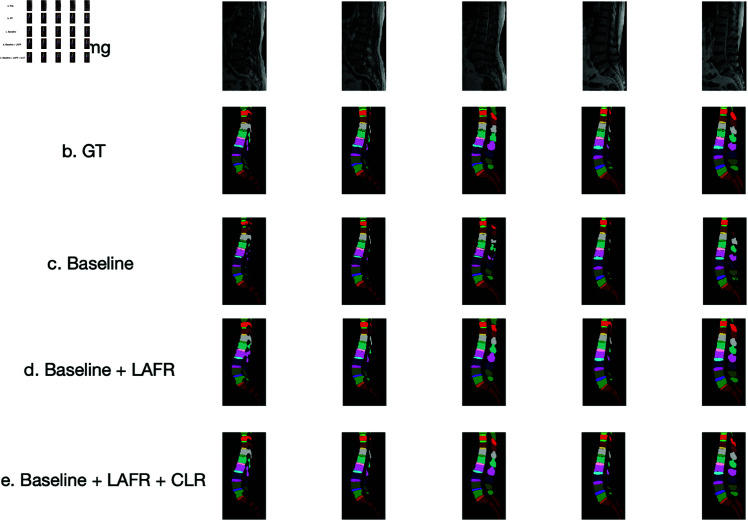
Qualitative results from the ablation study. CAFR-Net with LAFR and CLR modules exhibits superior boundary refinement and segmentation accuracy in low-contrast regions.

Fig 6 presents qualitative comparisons, illustrating the impact of each module on segmentation accuracy. The baseline model struggles with boundary inconsistencies and over-segmentation artifacts, particularly in low-contrast and complex regions. Introducing the LAFR module significantly improves boundary delineation, reducing segmentation discontinuities. The CLR module further enhances feature consistency, particularly in regions where subtle intensity variations make segmentation challenging. The full CAFR-Net model produces the most precise anatomical delineations, effectively mitigating misalignment and fragmentation issues.

These findings confirm that the LAFR module plays a crucial role in improving fine-grained spatial feature recalibration, while the CLR module enhances feature consistency and robustness to low-contrast anatomical variations. The integration of both modules yields state-of-the-art segmentation accuracy, validating the effectiveness of the proposed framework.

## 5 Conclusion

This paper introduces CAFR-Net, a novel segmentation framework that integrates global contextual modeling and local feature recalibration to enhance spinal MRI segmentation. The LAFR module refines spatially significant features, while the CLR module enforces anatomical consistency, particularly in low-contrast regions. Experimental results demonstrate that CAFR-Net achieves superior segmentation accuracy compared to existing CNN-based and Transformer-based methods.

**Limitations.** Although the SpineMRI dataset covers anatomically diverse regions and challenging low-contrast structures, its single-center origin limits generalizability to broader clinical contexts. The current evaluation does not include pathological conditions such as scoliosis or spinal tumors, nor does it incorporate external validation across multi-center cohorts. Future studies will assess generalizability by testing the model on public datasets such as VerSe Dataset [36], which encompass varied clinical environments and anatomical variations.

**Future work.** Future work will explore further improvements in computational efficiency to support deployment in resource-constrained or real-time clinical scenarios, including architectural simplification and module-level optimization. In addition, we aim to extend CAFR-Net to other anatomical structures and explore self-supervised learning strategies to enhance its generalization capability under limited annotation scenarios.

## Supporting information

S1 Appendix (DOCX)
