# Supplementary material for: CAFR-Net: A transformer-contrastive framework for robust spinal MRI segmentation via global-local synergy
Source: PLoS One. 2025 Jul 17;20(7):e0327642. doi: 10.1371/journal.pone.0327642 (PMC12270114; doi:10.1371/journal.pone.0327642)
Supplement: S1 Appendix — (DOCX) [file pone.0327642.s001.docx]

**S1 Appendix.** To rigorously evaluate the robustness of the selected loss weights, we performed a sensitivity analysis by individually perturbing each coefficient$\lambda_{1},\lambda_{2}$ and $\lambda_{3}$ while holding the others constant. The perturbation ranges ($\pm0.2$ for $\lambda_{1}$ and $\lambda_{2}$; $\pm0.05$ for $\lambda_{3}$) were determined based on prior work in multi-objective medical image segmentation [6,34] and a preliminary grid search on the validation set. A narrower adjustment window was applied to $\lambda_{3}$ due to its higher sensitivity in the contrastive regularization term [35], which influences feature alignment stability.

As shown in Table~\ref{tab:lambda_sensitivity}, the Dice and mIoU scores remained consistent across all tested configurations, with absolute deviations within $\pm0.$3 percentage points relative to the default setting ($\lambda_{1}$ = 0.5, $\lambda_{2}$ = 1.0, $\lambda_{3}$ = 0.1). These results confirm that the optimization process is resilient to moderate variations in the loss weighting scheme, supporting the empirical selection of these coefficients.

**Table5. Sensitivity analysis of loss weights on the SpineMRI dataset. Moderate perturbations of** $\boldsymbol{\lambda}_{\boldsymbol{1}}$**,** $\boldsymbol{\lambda}_{\mathbf{2}}$**, and** $\boldsymbol{\lambda}_{\boldsymbol{3}}$ **do not significantly affect segmentation performance.**

| **Loss Weights (**$\boldsymbol{\lambda}_{\boldsymbol{1}}$**,** $\boldsymbol{\lambda}_{\mathbf{2}}$**,** $\boldsymbol{\lambda}_{\boldsymbol{3}}$**)** | **DSC (%)** | **HD (mm)** | **mIoU (%)** |
| --- | --- | --- | --- |
| (0.3, 1.0, 0.1) | 91.82 | 3.66 | 88.97 |
| (0.7, 1.0, 0.1) | 91.89 | 3.60 | 89.05 |
| (0.5, 0.8, 0.1) | 91.75 | 3.71 | 88.84 |
| (0.5, 1.2, 0.1) | 91.93 | 3.58 | 89.06 |
| (0.5, 1.0, 0.05) | 91.90 | 3.59 | 89.02 |
| (0.5, 1.0, 0.15) | 91.87 | 3.63 | 88.94 |
| (0.5, 1.0, 0.1) | 92.04 | 3.52 | 89.31 |
